# Interactive causal diagram of habitat design impacts on behavioral health and performance in extreme environments

**DOI:** 10.1038/s41526-026-00618-9

**Published:** 2026-08-12

**Authors:** Mich Lin, Lu Chen, Katya Arquilla

**Affiliations:** 1 Department of Aeronautics and Astronautics, Massachusetts Institute of Technology, Cambridge, MA USA; 2 Department of Electrical Engineering and Computer Science, Massachusetts Institute of Technology, Cambridge, MA USA; 3 Smead Department of Aerospace Engineering Sciences, University of Colorado Boulder, Boulder, CO USA

**Keywords:** Engineering, Mathematics and computing

## Abstract

As crewed long-duration space exploration missions become increasingly Earth-independent and reliant on onboard technology, risks due to human-system incompatibility become critical to address. In a space habitat, establishing objective and meaningful relationships between the individual and the environment has been challenging due to multidirectional influences between system components. Feedback loops between behavioral health processes and outcomes complicate characterization of influence within the system. Yet, it remains crucial for habitat designers and stakeholders to trade design decisions and their associated risks. In this work, we have created a risk mapping of the impact of habitat design to behavioral health and performance outcomes in isolated, confined, and extreme environments. We leverage a Directed Acyclic Graph (DAG) to formalize habitat design parameters as powerful mediators between mission stressors and behavioral health outcomes. To represent the DAG accessibly, we created the *Human-Environment Connection & Interaction Atlas* (https://hecia.space), an interactive open-source platform. We conducted expert and user interviews to evaluate the underlying DAG and the usability of the tool. Herein, we present our novel development of a risk map that links habitat design and behavioral health, as well as an interactive visualization that can provide a basis for accessible communication of complex systems.

## Introduction

As of 2020, the NASA Human System Risk Board has tracked and managed myriad risks associated with human spaceflight to prepare for long-duration spaceflight missions to the Moon, Mars, and beyond^1^. NASA’s Human System Risk Board (HSRB) is currently tracking 29 risks that require mitigation for Lunar and Martian Design Reference Missions (DRMs), available via the NASA Human System Risks website (https://www.nasa.gov/hhp/human-system-risks/).

**Fig. 1:**
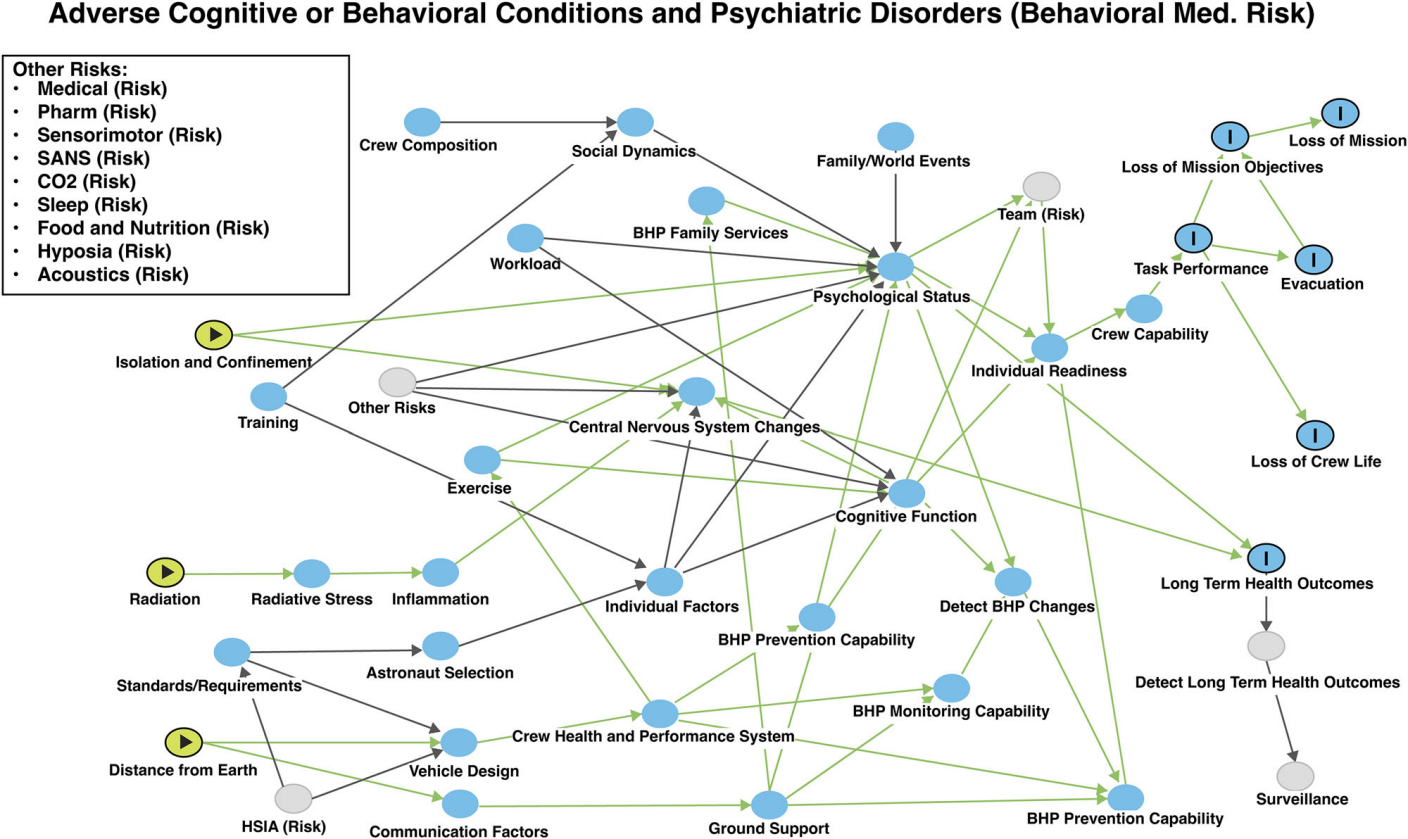
The Directed Acyclic Graph for the behavioral medicine risk. Adapted from the original DAG published by the NASA Human Systems Risk Board^22^.

To manage human spaceflight risks, several tools have been developed and implemented over the years. For instance, NASA has developed standards defining acceptable mission and lifetime health risks, as well as a risk mitigation analysis tool to evaluate the effectiveness of mitigation strategies^2^. In 2012, a visual tool was developed by Mindock and Klaus^3^, named the Contributing Factor Map. The CFM maps performance-influence factors in a vertical hierarchy from the most general levels at the top (i.e., Operations, Vehicle Design, and Human) to factors that directly influence performance. The CFM is an example of hierarchical node-connectivity mappings, which are widely used in human factors applications (e.g., work domain analysis^4^). Most recently, NASA has developed a visual representation of the compounding and synergistic risks to humans through a Directed Acyclic Graph (DAG) structure, which is a network map with arrows that point from one node to another (directed) and never form a feedback loop (acyclic). NASA DAGs visualize spaceflight-related risks to the human by connecting mission hazards, contributing factors, countermeasure capabilities, human system risks, and mission outcomes^5^. An example for one of the active HSRB risks can be found in Fig. 1. DAGs have been used in various scientific applications, including in epidemiology^6^ and public health^7,8^, to establish linkages between upstream events and downstream consequences. Compared with node-connectivity mappings (which communicate hierarchy and connection), DAGs can additionally provide information about causality or directionality. In complex systems, the DAG structure is favored for their ability to communicate the impact flow of decisions and their potential for network analysis^5^.

We sought to utilize the DAG structure to provide greater granularity toward mapping the impact of the habitat on behavioral health. Evidence from spaceflight and analog studies has shown that long-term isolation and confinement in an extreme environment have negative impacts on psychological and behavioral health^9–12^. For exploration missions with increased durations in isolation and confinement and high consequences of evacuation, the built environment becomes an even more salient mediating factor for crew health^13^. Indeed, past evidence suggests that habitat design has a close relationship to behavioral health and performance. For instance, the interior décor of spacecraft can affect wellbeing^14^; colors can be used as navigational aids in the microgravity environment^15^; and lighting systems need to provide both adequate task lighting and circadian entrainment^13^. In terrestrial contexts, theorists and practitioners from the fields of architecture and environmental psychology emphasize the importance of architecture on perception, cognition, behavior, and wellbeing^16,17^. As examples, the impact of the built environment on behavioral health has been acknowledged in Arctic urban settings^18^, post-disaster relief^19^, and prisons^20^.

The work described herein, the *Human-Environment Connection & Interaction Atlas* (HECIA), is focused on capturing the relationship between habitat design and behavioral health and performance. We present a DAG that maps the impact of mission design and the habitat environment to behavioral health outcomes. Importantly, we constructed this DAG to encompass a broad definition of isolated, confined, and extreme (ICE) environments—including spaceflight, and also Antarctic bases, submarines, post-disaster dwellings, war and conflict zones, and high density urban housing. Thus, we endeavor to include NASA HSRB definitions in our descriptions where applicable, and simultaneously expand upon the network architecture and existing definitions with extensive literature from both terrestrial and spaceflight contexts. This allows those working between engineering and design, those new to the discipline, or those outside of aerospace-adjacent paradigms, to more easily share their knowledge. We represent this DAG in a novel interactive platform, available at https://hecia.space (https://lin-shu-yu.github.io/spacearch). We detailed the development of the software platform in a previous publication^21^. Finally, we evaluated the DAG and the usability of the interface through subject matter expert interviews.

## Results

### Directed Acyclic Graph of habitat design impacts on behavioral health outcomes

Through an empirical approach with an extensive, iterative literature review, we created a DAG with 68 factors (nodes) and 167 connections (arrows). Every factor and connection is accompanied by a text description with relevant citations from spaceflight and ICE environment research. Factors are organized into groups, creating a layered structure for information management. To our awareness, the NASA DAGs are the only other form of causal mapping that addresses the human-habitat relationship in ICE environments. Thus, we make a comparison between the NASA DAGs and our DAG to clarify our contributions to the state-of-the-art. We expanded upon existing nodes within the NASA Narrative DAGs. We formalized 10 factors as *Designed Features* in our DAG, of which six are new factors related to “Vehicle Design” and “Environmental Conditions”. We formalized 14 distinct *Behavioral Health and Performance Outcomes* factors related to “Individual Readiness”, of which 11 are new factors. Notably, we link these categories of factors together in the same DAG structure meaningfully for the first time. For nodes that have a one-to-one mapping already existing within the NASA architecture^22^, we incorporated those definitions where applicable. We modified or supplemented them if the existing definition is spaceflight-specific to be applicable to general ICE environments. Some other factors without a one-to-one mapping are sub-nodes of existing NASA DAG nodes, and were extracted due to their particular salience to the human-environment relationship. Due to the level of detail required for our risk scope and to the intended applicability beyond spaceflight, we created many nodes that were not explicitly mentioned in the current NASA DAG definitions^22^. In the Supplementary Table 1, we provided a “translation guide” with greater detail about the matching of our factors to NASA DAG nodes or as sub-nodes, and noted where we deviated from existing NASA DAG architecture to represent salient factors in habitat–behavioral health considerations.

For all our nodes and connections, there exist accompanying text descriptions that feature cited references, which the current NASA Narrative DAGs do not have (although the Evidence Reports remain a wealth of information). With this strategy, we created an advantageous mix of both the visual appeal of DAGs and the utility of transparent, literature-informed evidence bases. The result is a comprehensive DAG structure of the impact of habitability on behavioral health and performance that can be standalone, or ported into the NASA DAG architecture.

### User interface platform

**Fig. 2:**
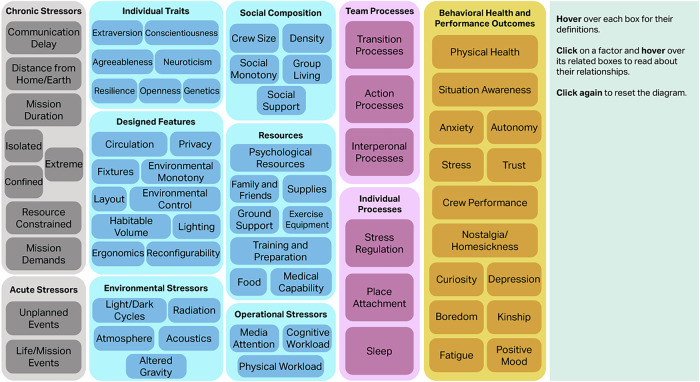
Drawing representation of the third layer of the Human-Environment Connection & Interaction Atlas platform. Darker boxes are interactable factors; users can (1) hover over them to read their definitions and see related factors, and (2) click on them, then hover over a related factor to read about their causal relationship. Lighter boxes represent organizational groupings from layer 2.

The *Human-Environment Connection & Interaction Atlas* (HECIA, available at https://hecia.space) is a three-layered framework that is hierarchically organized; the first layer consists of bins that cover items on the second layer, and so forth. These groupings serve as organizational headers in layer 3, shown in Fig. 2. Layer 3 is the final layer that visualizes all of the factors. Each layer is introduced incrementally to the user to aid comprehension. The layer navigation is facilitated via touchpad or mouse scroll, or via buttons in the bottom right corner of the browser page.

Each factor in layer 3 is an interactive node. Users can hover over the factor to read their definitions and see the first-degree connected factors, both before and after the causal flow. Users can also click on the factor, then hover over a connected factor to read about their causal relationship. Details on the software development process of the HECIA platform can be found in Lin et al.^21^.

### Usability and content evaluation interviews

The initial DAG was developed with a small team composed of experts in behavioral health and performance, human-system interaction, and space architecture. To evaluate the DAG and the user interface, we solicited input from experts and prospective users through one-hour semi-structured interviews. We conducted 13 interviews and coded 481 relevant comments representing design and/or engineering perspectives from NASA, academia, and industry. Around 35% of these comments were related to content, mostly concerning additions or connections to make in order to better represent risks to behavioral health and performance. Around 25% of the comments were related to the usage and operation of the user interface. Around 20% of the comments were suggestions for expanding the scope of the platform. Other coded instances outside of these groups included comments around the esthetics, organization of the layers, and quotes from the interviewees. We incorporated the majority of the content suggestions through creating, eliminating, or updating factors. Certain content and organizational suggestions were redirected into “usage contexts” instead, which we describe in the Discussion. Table 1 lists major suggested changes and our rationale for implementing or rejecting them.

**Table 1:** Interviewee feedback for the *Human-Environment Connection & Interaction Atlas* (HECIA), and rationale for implementation or rejection

Feedback	Implemented?	Rationale
CONTENT		
Include subclinical trends for negative mood	Yes	Supplemented “Anxiety” and “Depression” factors with subclinical considerations, but didn’t rename for brevity. Subclinical trends are important, especially for high-performing populations in operational environments.
Include off-nominal and contingency scenarios	Yes	Included under *Acute Stressors*. Off-nominal scenarios often arise in operational environments, and including them is important for design considerations.
Include positive psychology in *Behavioral Health and Performance Outcomes*	Yes	We added a separate factor for “Positive Mood”, which we determined as distinct from other behavioral health and performance outcomes. As considerations for crew wellbeing in spaceflight has historically for risk mitigation, this was an opportunity to represent a more holistic target where positive psychology is also emphasized. Additionally, existing mood and emotion literature suggest that positive and negative mood components have different pathologies and behavioral impacts^29,30^. Thus, as solutions for reducing negative mood may be different from those promoting positive mood, we included “Positive Mood” as a distinct factor in our graph.
Separate cognitive performance from "Crew Performance" into a separate factor	No	We preferred to incorporate cognition within "Crew Performance", as cognition affects all aspects of behavioral health and performance. Ultimately, the connective relationships between a new "Cognitive Performance" factor would not be a distinct map from that for "Crew Performance".
Include cultural composition	No	Culture is difficult to define, as there are many types (e.g., national, ethnic, religious, social, group). We chose to focus on functional impacts of culture that may mediate existing factors, for instance on "Group Living" standards or perception of "Density". This is where designers with specific insight into the cultural dynamic of a team can add interpretation.
Include operational pressure	Yes	Included under "Mission Demands", which was moved to *Chronic Stressors*. Operational pressure is distinct from "Media Attention", which had been included.
USABILITY		
Include a walkthrough tutorial	Yes	The landing page of HECIA is now a walkthrough tutorial, which can be exited and reopened. Most interviewees (12/13) missed the click-then-hover interaction to read about causal definitions between factors. As this is a crucial component of the network map, we created the walkthrough to increase usability and attention toward that functionality.
Add buttons for between-layer interaction	Yes	We included **+** and **-** buttons to aid navigation between the pages. Natural and non-natural scrolling directions could not both be accommodated, so buttons were a helpful inclusion. We added a **?** button which pulls up a text explanation of the platform and provides access to the walkthrough.
SCOPE		
Include optimal ranges for design parameters	Yes	For known and established factors (e.g., atmosphere, lighting), we included optimal and acceptable ranges documented in the literature. For certain factors (e.g., privacy, reconfigurability, medical capability), ranges are unknown or very nuanced. For those factors, we include resources for design guidance, where applicable.
Introduce hierarchy (e.g., numerical quantification, bolding lines) to indicate relationship strengths	No	We sought to avoid prescriptive usage of the tool. The field of behavioral health in ICE environments is still actively developing. The designers and stakeholders offer significant input and contribution, and we did not want to create a tool to replace the nuance of context and individual variation. We chose to add documented design ranges for known and established factors to be more comprehensive.

Symbols in the first column indicate the source(s) of feedback as follows: red (NASA), blue (industry), yellow (academia); circle (design/architecture), triangle (engineering/science).

## Discussion

We made several key novel contributions to formalizing the human-environment relationship for habitat design in ICE environments. Firstly, our DAG captures the impact of habitat design on behavioral health for both terrestrial and spaceflight applications. From an extensive literature review, we provided factor and causal relationship descriptions, allowing novices and experts alike to understand the context of the presented information. We introduced and described 14 distinct components of behavioral health and performance relevant in ICE environments. We additionally formalized a set of 10 distinct habitat design factors relevant to behavioral health, grouped under *Designed Features*. We utilized NASA terminology and incorporated NASA HSRB definitions where applicable^22^, and our rationale and harmonization guidance can be found in the Supplementary Table 1. In all these instances, we provided descriptions for the nodes and their connections through an extensive literature review spanning research from spaceflight to terrestrial contexts. By centering the descriptions on literature, we achieve two outcomes: (1) the informative quality of the DAG and the platform for non-experts and (2) traceable motivations for our definitions and connections.

Secondly, we developed an interactive, open-source design tool based on our DAG, available at https://hecia.space. HECIA has been designed as an interactive web interface to improve readability and accessibility, as compared to traditionally static text-based documentation. The user interacts with the DAG through layers of increasing complexity. These layers provide visual and graphical organization in a more intuitive and user-friendly manner, facilitating communication across stakeholders who may be unfamiliar with these topics. Finally, the interactive nature of the tool allows for designers, scientists, engineers, and researchers to extract relevant information and to explore impacts of design decisions in a clear and detailed manner. As habitat design and ensuring human wellbeing are inherently interdisciplinary challenges, inclusion of perspectives often not prioritized in aerospace is valuable and necessary.

Finally, we evaluated the usability of the HECIA platform and solicited feedback on the underlying DAG structure through interviews with subject matter experts and prospective users. We analyzed over 450 comments and consolidated the feedback for implementation into HECIA. Overall, both novices and experts alike found it a useful and engaging visualization of human-environment connections, with utility for education, communication of risks and design decisions, and informing habitat design activities. The interactive interface is available at https://hecia.space; the underlying code is in an open-source repository at https://github.com/lin-shu-yu/spacearch. Furthermore, HECIA was designed to be applicable to many Earth-based contexts, in addition to spaceflight. Through the inclusion of literature reference from psychology, anthropology, architecture, and voluntary and involuntary migration, this framework can be used to address challenges in ICE environments on Earth. Increasing the accessibility of a risk mapping platform for spaceflight can also add multidisciplinary expertise that strengthens off-Earth habitat design.

Several scope additions to the platform were suggested by interviewees. We determined that these were more appropriately represented as “contexts” through which to explore HECIA. These topics were not included as factors, as they affect the entire network map and how relative strengths and importance of relationships should be interpreted. With these discussions, we confirmed the necessity for experts to provide context into how these causal relationships are interpreted in the design process. A non-exhaustive list of contexts with brief descriptions are included in Table 2.

**Table 2:** Contexts in which designers may use the *Human-Environment Connection & Interaction Atlas* (HECIA) as a design tool

Context	Explanation
Specific destinations (Moon, Mars, Antarctica, submarine, war zone, etc.)	The destination affects the salience of chronic and acute stressors, which impacts all downstream factors. We envision this to be the most classical use of HECIA as a design tool, whereby designers have a specific design reference mission or environmental context in mind.
Extravehicular activity (EVA)	EVA can be orbital or on a planetary surface. One could consider the EVA habitat (in this case, spacesuits, rovers, and/or other supporting equipment) as the context in which to interpret HECIA. The presence of EVA in mission planning, which restricts many factors (e.g., food, habitable volume, training and preparation) is currently represented under *Mission Demands*.
The degree of realistic risk	When considering operational analog, there may be a varying degree of fidelity or realism as compared to spaceflight. The intrinsic knowledge that lives are not truly at stake may distort the relational strength of many of the upstream factors to behavioral health outcomes. Certain mission stressors (e.g., deep space, “dark” telecommunications) will likewise distort these relationships, whereby the heightened risk makes human-environment relationships more important or consequential.

However, limitations also exist in our approach. In any representational tool or model, the fidelity and depth of the knowledge is traded-off against usability and readability. Similarly, the content of each factor is limited to the depth required for comprehension by a wide range of users with variable expertise, the time and effort required to utilize this tool, and the familiarity of the topic to the intended audience. The number of factors were also constrained to fit on a full-screen, browser-sized page when viewed from a laptop. Heuristics, which designers rely on, are also underrepresented in a literature-driven knowledge map like this. DAGs capture acyclic relationships, but humans also affect the systems with which they interact. While the current representation is amenable to the design process—where earlier decisions impact habitat design, which in turn impacts behavioral health and performance—feedback loops in which behavioral health impacts upstream factors were not captured here. Finally, the evaluation of the DAG and HECIA platform thus far represents a sliver of the underlying truth, and a limited portion of the existing knowledge and opinions. Unlike the NASA Narrative DAGs, whose authority relies on informed risk custodians and an extensive development process with the HSRB board and broader community, our DAG process is lightweight. While this development process has its own advantages, it is more prone to quality issues. Similar to the NASA HSRB, we also welcome comments from the greater community to refine our DAG.

Future work in DAG validation and evaluation will add valuable insight to habitat design domain. Input and verification of the DAG, its nodes, and its connections from the community will be valuable, especially as this tool is now publicly available. Validation of the DAG structure is a natural next step^23^; however, we expect validation to be an iterative and lengthy process, as gathering sufficient data to formulate coherent causal relationships is especially challenging in behavioral and psychological research^24^. The necessity of producing a validated, representative DAG for risk assessment and reduction further motivates fundamental research on behavioral and cognitive health. Verification and validation of the user interface will also provide further insight into usability, and prepare the tool for trustworthy stakeholder adoption.

Embedding hierarchy into HECIA was a recurring theme throughout the interviews. Suggestions were both qualitative—introducing dashed lines for secondary connections, bolding stronger causal connections; and quantitative—including effect sizes, adding numerical values to upstream risks and calculating downstream impacts. To do so, we would need to take HECIA as a sort of “ground truth” that represents the reality; however, it only represents a portion of documented literature. Human behavior is also notoriously difficult to model and predict^25^. We have chosen not to create a prescriptive tool at the risk of overextending our confidence in the state of research. Additionally, inter-individual differences permeate across physiology, psychology, and behavior^26^, potentially rendering inapplicable any hierarchical mapping that generalizes and emphasizes certain risks and impacts over others. However, we acknowledge the desire for additional sources of knowledge. Toward this end, we hosted a series of workshops where participants can create their own DAGs using the HECIA framework to capture their lived experience in spaceflight and operational analogs, or as habitat designers. We received positive feedback regarding the utility and user experience of the tool, and verified our DAG and HECIA’s interactivity as productive mechanisms for capturing personal and heuristic knowledge about habitability in ICE environments. A similar extension to capture the lived experiences of those in terrestrial settings (e.g., high-density urban housing) could be conducted using the HECIA platform.

## Methods

We developed a DAG and an interactive platform that addressed key opportunities posed by the habitat-design specific knowledge, comprehensibility, and legibility gaps within existing methods, namely the standards^2^, factor mapping^3^, and NASA DAGs^27^. We describe the habitat design–behavioral health DAG and the associated interactive user interface as two separate efforts in this section.

### Directed Acyclic Graph development

Through an extensive and iterative literature review process, we created a DAG that maps aspects of an ICE environment to behavioral health and performance outcomes, emphasizing the relationship between habitat design and behavioral health. We aimed to maintain portability to allow harmonization with the NASA risk mapping context, while adding detail to the habitat-human connection and extending applicability to ICE environments beyond spaceflight.

The initial network graph was developed with a small team composed of experts in behavioral health and performance, human-system interaction, and space architecture. To capture the appropriate set of factors for inclusion in our DAG, we focused the scope of knowledge on habitat-related risks impacting behavioral health and performance. Aligned to the NASA HSRB DAG guidance^27^, we represented the Spaceflight Hazards (Altered Gravity, Radiation, Isolation and Confinement, Hostile Closed Environment, Distance from Earth) in some form within our DAG. However, since HECIA extends beyond spaceflight applications, we include other variables as starting points for causal flows. We organized our factors chronologically, such that the factors under *Mission Parameters* are typically decided first.

Rather than NASA’s “Mission Level Outcomes” (Task Performance, Evacuation, Loss of Mission Objectives, Loss of Crew, Loss of Mission, Flight Recertification, Long Term Health Outcomes), the causal flows within our DAG end at a factor in *Behavioral Health and Performance Outcomes*. We had initially included “Mission Level Outcomes” as the ending point of causal flows. However, we observed that virtually all *Behavioral Health and Performance Outcomes* factors justifiably contribute toward “Mission Level Outcomes,” which is logical given that these are the common ending points for NASA DAGs. However, considering the focus of this work, we ultimately chose not to explicitly include the NASA ‘Mission Level Outcomes’. Further, we were restricted in space in the web interface, and we chose to prioritize other functions (e.g., the extended textual explanation). In comparison with the NASA DAG architecture, we envision that the *Behavioral Health and Performance Outcomes* would be one node (or sub-nodes of “Individual Readiness”) that contributes toward the Mission Level Outcomes.

In between starting and ending nodes, there are myriad factors that mediate and affect the flow of causality. In the NASA DAGs, these are “Contributing Factors”; we categorize our contributing factors as *Mediator Variables* and *Processes*, to emphasize the temporal nature of these influences underlying behavioral health and performance changes. Within the two categories of contributing factors, we used additional groupings as visual and cognitive aids.

Our descriptions, for both the nodes and the edges, typically offer greater detail than the NASA definitions, with supporting literature citations. Many of the factors (24/68) we included have a corresponding factor within the NASA DAG architecture (i.e., Appendix A aka “DAGtionary” in ref. ^22^). In the case of a one-to-one mapping, we strove to include the NASA DAG definition as part of our definitions. Of the factors that do not have a one-to-one mapping, many are sub-nodes (explicitly defined in the NASA DAG node definition) or super-nodes of NASA DAG nodes and can be easily integrated into the existing NASA DAG architecture. However, there are a significant amount of factors we have developed that are not explicitly defined as a part of a NASA DAG node. Together, these deviations comprise the contributions we make in this work: (1) adding fidelity to the subset of vehicle design parameters and behavioral health and performance considerations in a DAG structure; (2) extending the applicability of this DAG into non-spaceflight contexts. We include a “translation guide” in Supplementary Information, where we summarize the overlaps and deviations from existing NASA terminology. For new nodes, we reference NASA nodes within which we would suggest our factors be incorporated.

### User interface development

A design tool optimally leverages our DAG to positively impact behavioral health and performance in ICE environments. To ensure the usability and accessibility of the information presented in our DAG, we created an accompanying user interface to enable informed design of technologies for ICE environments. We utilized GitHub (GitHub, Inc., San Francisco, CA, USA), an open-source software development platform, to build our codebase for the interactive platform. The code comprised HTML, JavaScript, and CSS, and is available at https://github.com/lin-shu-yu/spacearch. We visualized the factors and connections in the last layer of a three-layer framework, and the previous two layers as broader conceptual bins.

We assigned user interaction gestures to distinct categories of information captured within the DAG. Hovering over a factor highlights the first-level causal connections for that factor and its definition on the right-hand static text panel. This information disappears when the user is not actively hovering over a factor. Clicking on a factor “sets” the hovering view, whereby the user can hover off of that factor and the information would not disappear. Clicking on a factor, then hovering over a connected factor shows the text explanation for the causal relationship on the right-hand static text panel.

### Subject matter expert and prospective user interviews

One-hour semi-structured interviews about completeness, representativeness, and usability were conducted with subject matter experts and prospective users (*N* = 13). We sought feedback about the utility and faithfulness of the DAG, and the usability of the web interface. Prospective users represented the tool’s intended audience (e.g., students, design/architecture/aerospace experts who may be novices to one of the other areas but are specifically interested in habitat design). We assigned participants a primary institutional affiliation (NASA, academia, or industry) and one or both disciplinary groupings (architecture/design; engineering/science) based on their self-reported professional title. The participants were primarily affiliated with NASA (1 architect/designer, 3 engineers/scientists), academia (1 architect/designer, 5 engineers/scientists), and industry (2 architects/designers, 1 who was both an architect/designer and engineer/scientist). The sample population was derived using a purposeful stratified sampling method^28^, wherein key areas of domain knowledge and their relative representation in the field guided the proportion of interviewees. Interviewees were identified via referral from the initial expert team or via chain referral from other interviewees. We coded de-identified interview transcripts for themes using Atlas.ti (ATLAS.ti Scientific Software Development GmbH, Berlin, Germany).

All procedures described herein conformed to US and UN regulations governing the treatment of human research participants, and complied with the American Psychological Association Code of Ethics and tenets of the Declaration of Helsinki. All protocols were approved and monitored by the MIT Committee on the Use of Humans as Experimental Subjects (Exempt Protocol #E-5793). Informed consent was obtained from each participant.

## Supplementary information

Supplementary information

## Data Availability

The datasets generated and analyzed during the current study are not publicly available due to Institutional Review Board limitations but are available from the corresponding author on reasonable request.

## Code availability

The underlying code for this study is available in GitHub and can be accessed via this link: https://github.com/lin-shu-yu/spacearch.
