# Supplementary material for: Interactive causal diagram of habitat design impacts on behavioral health and performance in extreme environments
Source: NPJ Microgravity. 2026 Aug 12;12:68. doi: 10.1038/s41526-026-00618-9 (PMC13470023; doi:10.1038/s41526-026-00618-9)
Supplement: Supplementary file 1 [file 41526_2026_618_MOESM1_ESM.pdf]

Supplementary Table 1: 'Translation guide' between our definitions (available via https://github.com/lin-shu-yu/spacearch or https://hecia.space) and Appendix A aka 'DAGtionary' in [1]. Uncolored cells represent factors where there exists a one-to-one mapping. We include NASA definitions in our factor descriptions when applicable. Green colored cells represent factors that are sub-nodes (explicitly defined in the NASA DAG factor definitions) or super-nodes. These factors can be easily integrated into the existing DAG architecture. Blue colored cells represent factors which are not explicitly defined in the current NASA DAG architecture.

	**NASA Equivalent**		
**Factor Name**	**Node**	**Sub-node of...**	**Notes**
Acoustics		Environmental Conditions	We extract acoustics from the overall environmental conditions as it's a key habitability concern [2, 3].
Action Processes			We separated key processes within teaming to provide granularity [4], as team and social processes are critical for behavioral health [5]. The NASA definitions do not provide a node for team processes, but when harmonizing, we would tentatively suggest this to be a sub-node of 'Team Functionality'.
Agreeableness		Social Dynamics	We separated personality traits according to the Big Five model as aspects of personality have different influence toward behavioral health and performance factors.
Altered Gravity	Altered Gravity		We included NASA's definition in our description.
Anxiety			When harmonizing, we would advise this to be a sub-node of 'Individual Readiness'.
Atmosphere	Atmospheric Conditions		We included NASA's definition in our description.
Autonomy			When harmonizing, we would advise this to be a sub-node of 'Individual Readiness'.
Boredom			When harmonizing, we would advise this to be a sub-node of 'Individual Readiness'.

Factor Name	Node	Sub-node of...	Notes
Circulation			When harmonizing, we would advise this to be a sub-node of 'Vehicle Design'.
Cognitive Workload		Workload	We included the partial definition related to the cognitive aspect of workload in our description.
Communication Delay	Communications Delay		We included NASA's definition in our description.
Confined		Isolation and Confinement	We considered NASA's definition when creating our description. Ours is extended and we consider *Confinement* into a separate factor as it has a different risk pathway.
Conscientiousness		Social Dynamics	We separated personality traits according to the Big Five model as aspects of personality have different influence toward behavioral health and performance factors.
Crew Performance			We included Crew Performance as a large umbrella term for considering traditional individual, team, and task performance. However, the Behavioral Health and Performance Outcomes factors are largely distinct from existing NASA terminology as we emphasized numerous aspects of behavioral health that were not explicitly represented in the NASA Narrative DAGs. The existing and traditional focus of "performance" is represented in this term. When harmonizing, we would tentatively suggest this to be a super-node of 'Individual Readiness' and 'Team Functionality'.

Factor Name	Node	Sub-node of...	Notes
Crew Size	Crew Size		We shortened this definition to “The total number of people on a team.” to apply to ICE environments generally.
Curiosity			When harmonizing, we would advise this to be a sub-node of ‘Individual Readiness’.
Density			When harmonizing, we would advise this to be a sub-node of ‘Vehicle Design’.
Depression			When harmonizing, we would advise this to be a sub-node of ‘Individual Readiness’.
Distance from Home/Earth	Distance from Earth		We did not implement NASA’s definition. Instead, we used “The distance (temporal and physical) from one’s location to one’s ‘home’.” as both the physical and temporal aspects are relevant for behavioral health in the contexts of interest (e.g., time zone difference for separated families).
Environmental Control	Environmental Control		We adapted NASA’s definition, leaving out ‘operates’ to apply to ICE environments generally.
Environmental Monotony			When harmonizing, we would advise this to be a sub-node of ‘Vehicle Design’.
Ergonomics			When harmonizing, we would advise this to be a sub-node of ‘Vehicle Design’.
Exercise Equipment	Exercise Hardware		We adapted NASA’s definition, leaving out ‘that enables successful mission task performance’ to apply to ICE environments generally.
Extraversion		Social Dynamics	We separated personality traits according to the Big Five model as aspects of personality have different influence toward behavioral health and performance factors.

Factor Name	Node	Sub-node of...	Notes
Extreme			We extended the hostility of the environment beyond NASA's definition, as extremeness is very diverse and variable across ICE environments. When harmonizing, we would advise this to be equivalent to 'Hostile Closed Environments'.
Family and Friends			We specify *Family and Friends* since these influences are common to those in ICE environments, and can even more saliently impact behavioral health and performance in cases of involuntary displacement. We did not use NASA's definition, as that was primarily concerned with the impacts of family wellbeing (both positive and negative), and we refer to this factor as a noun in our structure. When harmonizing, we would advise this be an equivalent node of 'Family Effects'
Fatigue	Fatigue		We included NASA's definition in our description.
Fixtures			When harmonizing, we would advise this to be a sub-node of 'Vehicle Design'.
Food	Food System		We included NASA's definition in our description.
Genetics		Non-Modifiable Individual Factors	We specify *Genetics* as a simplified, but important, representation of how non-modifiable biological factors influence behavioral health and performance outcomes.
Ground Support	Ground Support		We did not use NASA's definition, as it was spaceflight specific. Instead, we opted for "Support that comes from an external resource (usually a consistent community of trained staff) for the crew".

Factor Name	Node	Sub-node of...	Notes
Group Living		Social Dynamics	We extracted *Group Living* to emphasize its importance to behavioral health and performance outcomes.
Habitat Volume	Net Habitable Volume		We adapted NASA's definition for our description, removing mention of 'spacecraft' and including 'access' as a criteria for habitable volume.
Interpersonal Processes			We separated key processes within teaming to provide granularity [4], as team and social processes are critical for behavioral health [5].The NASA definitions do not provide a node for team processes, but when harmonizing, we would tentatively suggest this to be a sub-node of 'Team Functionality'.
Isolated		Isolation and Confinement	We considered NASA's definition when creating our description. Ours is extended and we consider *Isolation* into a separate factor as it has a different risk pathway.
Kinship			When harmonizing, we would advise this to be a sub-node of 'Individual Readiness'.
Layout			When harmonizing, we would advise this to be a sub-node of 'Vehicle Design'.
Life/Mission Events			NASA includes a definition for 'Family/World Events', but here we would include those and mission events (e.g., launch, docking).
Light/Dark Cycles		Environmental Conditions	We extracted the external lighting conditions from 'Environmental Conditions' as altered daylight conditions are especially relevant to ICE environments (e.g., Ant/Arctica, polar circle, submarines).

Factor Name	Node	Sub-node of...	Notes
Lighting		Environmental Control	We extracted *Lighting* as a factor due to its significance in habitat design. When harmonizing, this could also be considered to be a sub-node of 'Vehicle Design'.
Media Attention			Media attention is typically positive within the spaceflight context, as public engagement and excitement are prevalent. However, in disaster or refugee contexts, this can have both positive and negative impact. We decided to include it as a factor not currently represented in the NASA architecture. When harmonizing, we would tentatively suggest this be a sub-node of 'Family/World Events'.
Medical Capability	Medical Monitoring Capability, Medical Diagnostic Capability, Medical Treatment Capability		In our description, we include references to the three NASA Medical capabilities (Monitoring, Diagnostic, and Treatment). When harmonizing, we would advise this to be a super-node of these capabilities.
Mission Demands			We distinguish Mission Demands (chronic) from Mission Events (acute). When harmonizing, we would tentatively advise this to be a sub-node of 'Operations Design'.
Mission Duration	Effective Mission Duration		We adapted NASA's definition for our description, generalizing to time away from 'origin' instead of 'surface of Earth'.
Neuroticism		Social Dynamics	We separated personality traits according to the Big Five model as aspects of personality have different influence toward behavioral health and performance factors.

Factor Name	Node	Sub-node of...	Notes
Nostalgia/ Homesickness			When harmonizing, we would advise this to be a sub-node of 'Individual Readiness'.
Openness		Social Dynamics	We separated personality traits according to the Big Five model as aspects of personality have different influence toward behavioral health and performance factors.
Physical Health		Physical State	We specified health to emphasize the temporal nature of the physical state, as health is more than a snapshot in time; our description includes elevated risk to long-term health outcomes. When harmonizing, we would tentatively advise this to be a sub-node of 'Physical State'.
Physical Workload		Workload	We included the partial definition related to the physical aspect of workload in our description.
Place Attachment			The NASA definitions do not provide a node with similar consideration. We consider place attachment to be a core function of human-habitat interaction. When harmonizing, we would tentatively suggest this to be a sub-node of 'Individual Readiness'.
Positive Mood			When harmonizing, we would advise this to be a sub-node of 'Individual Readiness'.
Privacy	Privacy/Team Space		We chose to use the original definition of privacy that the NASA-STD-3001 definition is derived from.
Psychological Resources	BHP Intervention Capability, BHP Family Services		We did not adapt NASA's definition as it was spaceflight-specific, but we considered it in our description.

Factor Name	Node	Sub-node of...	Notes
Radiation	Radiation		We included NASA's definition in our description.
Reconfigurability			When harmonizing, we would advise this to be a sub-node of 'Vehicle Design'.
Resilience		Social Dynamics	We consider resilience as a personality trait, which is incorporated under NASA's definition for 'Social Dynamics'.
Resource Constrained	Resupply		We included NASA's definition in our description. We use "resource constrained" to also include considerations of the lack of resources in the initial state.
Situation Awareness			When harmonizing, we would advise this to be a sub-node of 'Individual Readiness'.
Sleep			The NASA definitions do not provide a node for individual processes, but when harmonizing, we would tentatively suggest this to be a sub-node of 'Sleep Deficiencies'.
Social Monotony			When harmonizing, we would advise this to be a sub-node of 'Social Dynamics'.
Social Support	Social Support		We included NASA's definition in our description.
Stress	Stress		We partially included NASA's definition in our description, leaving out 'stress hormones' for concise scope.
Stress Regulation			The NASA definitions do not provide a node for individual processes, but when harmonizing, we would tentatively suggest this to be a sub-node of 'Individual Readiness'.

Factor Name	Node	Sub-node of...	Notes
Supplies		Resupply	For completeness, we use *Supplies* to encompass equipment and consumables that were not directly identified in our architecture. For this reason, there is no one-to-one mapping toward the NASA architecture. When harmonizing, we would tentatively advise this be a sub-node of 'Resupply' or otherwise discarded as the NASA architecture likely offers more fidelity in this instance.
Training and Preparation	Training		We adapted NASA's definition for our description and extended it to general ICE environments.
Transition Processes			We separated key processes within teaming to provide granularity [4], as team and social processes are critical for behavioral health [5].The NASA definitions do not provide a node for team processes, but when harmonizing, we would tentatively suggest this to be a sub-node of 'Team Functionality'.
Trust			When harmonizing, we would advise this to be a sub-node of 'Individual Readiness'.
Unplanned Events	Anomalous Events		We included NASA's definition in our description.

References

[1] Antonsen, E.L., Monti, A., Charvat, J., Connell, E.S., Reynolds, R.J., Abukmail, A., Marotta, K., Brown, C.: Directed acyclic graphs: A tool for understanding the nasa human spaceflight system risks-human system risk board (2022). NASA/TP-20220015709

[2] Pearson, A.O., Grana, D.C.: Preliminary Results from an Operational 90- Day Manned Test of a Regenerative Life Support System., (1971)

[3] Harrison, A.A., Caldwell, B., Struthers, N.J.: INCORPORATION OF PRIVACY ELEMENTS IN SPACE STATION DESIGN. NASA (1988)

[4] Marks, M.A., Mathieu, J.E., Zaccaro, S.J.: A temporally based framework and taxonomy of team processes. The Academy of Management Review **26**, 356 (2001)

[5] Dev, S.I., Dotson, V., Mulavara, A., Nelson, G.A., Schorn, J.M., Whitmire, A.M., Whiting, S.E., Williams, T.J.: Evidence report: Bmed. NASA (2023)
